# Commentary: we can tell where it hurts, but can we tell where the pain is coming from or where we should manipulate?

**DOI:** 10.1186/2045-709X-21-35

**Published:** 2013-10-21

**Authors:** O’Dane Brady, Scott Haldeman

**Affiliations:** 1 World Spine Care, PO Box 49, Mahalapye, Botswana; 2 University of California, Irvine, USA; 3 University of California, Los Angeles, USA

**Keywords:** Chiropractic, Spinal manipulative therapy, Spine palpation, Clinical decision making

## Abstract

The shared decision making process has become increasingly important in the management of spinal disorders where there remains a variety of treatment options. Spinal manipulative therapy (SMT) is often recommended as a conservative option by evidence based clinical practice guidelines and a treatment modality frequently utilized by chiropractors and other clinicians who offer SMT to their patients. This article serves as a commentary to a review of the methods that are often used by chiropractors to determine the site for applying their manipulative intervention. Though it may be easy to criticize any review of this type of literature and point out shortcomings there are strong take away messages for the clinician interested in employing SMT as a part of their treatment protocol. Most notably, clinicians can be reassured that a history on the localization of pain, tissue palpation, provocative testing, range of motion testing and the demonstration by the patient of the locus and description of pain have reasonable consistency between observers. What this paper does not inform us on is the nature of the lesion causing the pain or where the manipulation should be applied to obtain the best outcome.

## Commentary

Clinicians faced with a patient with spinal pain must make a number of decisions prior to offering treatment options for the patient to consider in the shared decision making process [1]. This process is increasingly recommended before a treatment begins and is assumed to result in the most effective and patient preferred outcomes. Shared decision making is increasingly important in an era where there are over 200 treatment approaches for the management of spinal pain with very few of them having any real evidence of effectiveness [2].

The decisions that are part of this process are:

(1) Are there any red flags for serious pathology?

(2) Is there any evidence for neurological compromise?

(3) Where is the spinal pain and can the location of the pain be accurately determined?

(4) What structure or physiological process is causing the pain?

(5) What treatment is likely to improve the symptoms?

The first two components of this decision making process are incorporated in all guidelines on the management of spinal pain. However, despite being essential to the diagnostic process, there remains considerable concern about the reliability of many of the procedures used to rule out serious pathology and neurological deficits given the high false positive rates when these signs are used in isolation. What is clear, is that this process requires that every clinician who treats patients with spinal pain take a complete history and physical examination of the patient and correlate their findings with other clinical tests when necessary, before proceeding in the decision making process.

One of the few treatment approaches which have a reasonable level of research evidence of effectiveness and which is usually listed in widely recommended guidelines for the management of neck and back pain is manipulation [3,4]. Although spinal manipulation is offered by a number of professions chiropractors have been most closely identified as providers of spinal manipulative therapy (SMT) which has been considered the ‘mainstay’ of the profession for over 100 years [5]. The primary diagnostic tool that is stressed at chiropractic educational institutions and in most post graduate manipulation skills courses is the honing of exceptional palpation skills. Chiropractors spend entire semesters palpating each other and then use these skills when they begin treating patients in the clinical setting. Most of the procedures that are taught in the colleges have been an integral part of chiropractic and manual therapy throughout its history and includes the location of tender areas or provocation of pain on specific manoeuvres, static and motion palpation and range of motion testing. The generally accepted goal of palpation is to identify the source of pain, the so called subluxation or the level of spinal restriction and to determine where and what type of manipulative, adjustive, or manual therapy should be considered.

This process is complicated by the multiple undergraduate and post-graduate courses that promote very specific and complex diagnostic procedures to identify the site of manipulation. These procedures are often named after a particular method or system of spinal manipulation, not only in chiropractic but also in physical therapy and osteopathic courses. In addition there are a host of mostly controversial yet widely promoted and taught, instruments and diagnostic techniques used for detecting the site of the manipulative lesion. These methods include x-rays, skin temperature, complex muscle testing, skin conductance, and electrodiagnostic procedures that along with other diagnostic methods are often associated with some form of commercial or practice building incentive.

The article by Triano et al. in this issue of the journal [6] attempts to assess the current research that supports the methods that are used by chiropractors to determine the site for applying their manipulative intervention. They have done an intensive search of the literature to find research that has looked at this question and have adequately assessed the literature. It is easy to criticize any review of this type of literature and the shortcomings in this article are easily determined and mostly pointed out by the authors. These include the heterogeneity of the available literature, the fact that much of the evidence is conflicting or inconclusive and that most of the studies have been done on young healthy subjects rather than a wide spectrum of symptomatic patients. One should also consider the fact that almost all the recommendations are based on inter-observer reliability with little consideration as to whether the findings are meaningful or actually result in greater outcomes following treatment. Despite these criticisms it is possible to take a few important points away from this paper that can impact the daily practice of chiropractors and clinicians involved in spine care. The readers of this paper can take some reassurance that a history on the localization of pain, tissue palpation, provocative testing, and range of motion testing remain an integral part of the diagnostic process. Furthermore the authors have concluded that static and motion palpation and the demonstration by the patient of the locus and description of pain have reasonable consistency between observers. This therefore suggests that they may be an important part of chiropractic practice or for that matter the examination process by any clinician who is anticipating offering manual or manipulative treatment to a patient. However, further research remains necessary given the lack of strong evidence for palpation in localizing the site of care.

The authors of this review have also concluded that the literature does not provide convincing evidence for the use of x-rays or any diagnostic device in determining the site of care. It is therefore important for the clinician to keep in mind that, in the absence of a history to suggest serious pathology, x-rays to seek out postural or biomechanical changes within the spine are unlikely to change the course of the proposed treatment or add any additional information to the overall clinical picture and only serve to perpetuate the overutilization of diagnostic imaging procedures utilized for the assessment of spinal pain [7]. It is also evident from this paper that specific named systems of determining where to manipulate often taught by charismatic instructors including manual muscle testing or novel devices used for the same purpose or to generate income (e.g. surface EMG and thermography) have not been validated and are not worth considering.

The authors point out, that for some of the most widely used procedures such as simple palpation, there remain considerable weaknesses in the literature and this should be kept in mind when clinicians are interpreting their clinical findings. Of greater importance, however, is the fact that none of the studies that these authors quote have tested what the meaning of the diagnostic procedures are or how they should be interpreted. The fact that tenderness, range of motion or skin characteristics are reasonably reproducible between observers does not give us any information about what is actually being palpated, what lesion or tissue is causing the pain or anything about whether the proposed manipulation or adjustment actually changes the positive findings noted in the examination. It also does not tell us whether any change in these findings relate to patients symptoms or disability. Furthermore, in the absence of any gold standard there is no means of determining the sensitivity or specificity of the tests in assessing the patient’s complaint.

These weaknesses, however, are not the sole problem of chiropractors. The same can be said for many of the diagnostic tests that are currently being used by family physicians, neurologists, orthopaedic or neurosurgeons, physical therapists, osteopathic physicians and the increasingly growing number of health care professionals that offer care to people with spinal pain. As the authors of this paper point out there is currently no gold standard for the assessment of spinal pain. The expensive and painful assessment procedures often used by medical specialists including pain provocation or blocking procedures such as discography, facet injections etc. have failed to convincingly isolate the painful lesion [8]. Furthermore the inter-observer reliability of some of the standard medical procedures including MRI have reliability estimates that are similar to those described for manual diagnostic procedures.

Given the shortcomings of diagnostic tests for non-specific spine pain there is a growing attempt on the part of some clinicians to seek alternative approaches to the assessment of patients. One tool that is gaining some attention in the management of spinal pain is the use of clinical prediction rules to identify patients most likely to respond to a specific treatment approach. These rules classify patients into sub-groups based on the clinical examination rather than a presumed identification of the painful lesion and have shown some promise [9] in identifying the patients likely to respond to specific treatment approaches. However, this approach still requires further refinement before it can be generally accepted.

The assumption in this article by Triano et al. is that the manipulation will be applied to the manipulable lesion identified in the testing. This question has to be considered by any clinician who may wish to apply SMT to an unrelated area of the spine (i.e. SMT to the thoracic spine for the management of neck pain or limiting SMT to the cervical spine irrespective of the presenting complaint and examination findings). It is clear that none of these studies can be considered to justify such an application.

Finally, these study findings should not be considered to be solely of interest and use by chiropractors. Any spine care provider who may be interested in employing SMT or referral of a patient with spinal pain to a chiropractor should consider using these examination tools. Educational institutions and examination boards should also be observant of the study findings and should make every effort to avoid teaching or examining chiropractors or any professional who is learning to apply SMT on those procedures that have no reasonable scientific support.

## Competing interests

Both authors declared that they have no competing interests.

## Authors’ contributions

All authors critically appraised the review by Triano and colleagues. OB wrote the initial draft of the commentary. SH provided suggestions for changes to the draft which were then incorporated. The final draft was approved by all authors.
